# Detection of antibodies to *Toxoplasma gondii* in wild animals in Brazil

**DOI:** 10.1186/1678-9199-20-41

**Published:** 2014-09-09

**Authors:** Rodrigo Costa da Silva, Gustavo Puglia Machado, Tatiane Morosini de Andrade Cruvinel, Ciro Alexandre Cruvinel, Helio Langoni

**Affiliations:** Departamento de Higiene Veterinária e Saúde Pública, Faculdade de Medicina Veterinária e Zootecnia (FMVZ), Universidade Estadual Paulista (UNESP), Distrito de Rubião Júnior, s/n, Botucatu, SP CEP 18618-970 Brasil; Veterinary Hospital, University Center of Rio Preto (UNiRP), São José do Rio Preto, São Paulo, Brazil; Veterinary Consultant, Bady Bassit, São Paulo, Brazil

**Keywords:** *Toxoplasma gondii*, Natural infection, Antibodies, Wild animals, MAT

## Abstract

**Background:**

Toxoplasmosis is a worldwide zoonosis caused by an obligate intracellular protozoan parasite, *Toxoplasma gondii*, that affects all warm-blooded animals, including wild animals. The increased number of cases of parasitic infections is mainly due to the destruction of environmental conservation areas, which is driving wild animals out of their habitats and towards urban areas. In this study, the occurrence of *T. gondii* infection was investigated by the modified agglutination test (MAT) in 26 different species of run over and injured wild animals that were treated at a Brazilian university veterinary hospital, from June 2007 to August 2008.

**Findings:**

Of the studied animals, six (23.1%; CI95% 11.1-42.2%) had *T. gondii* antibodies, with titers equal to 10 (4; 66.7%) and 40 (2; 33.3%). The species *Pseudalopex vetulus*, *Cerdocyon thous*, *Hydrochoerus hydrochaeris* and *Tapyrus terrestris* had titers of 10, while *Alouatta caraya* and *Puma concolor* had titers of 40. There was no significant association regarding age, gender or purpose of care (*p* > 0.05).

**Conclusions:**

Carnivorous, herbivorous and omnivorous wild animals are potential sentinels of human toxoplasmosis, especially when wild felids are present, maintaining the environmental contamination.

## Findings

Toxoplasmosis is one of the most common parasitic zoonoses throughout the globe. It is caused by *Toxoplasma gondii*, an intracellular protozoan parasite, that has a heteroxenous life cycle and infects all warm-blooded animals (pets, wild and farm animals) and humans [1–3]. Wild and domestic felids are the definitive hosts, which release parasite oocysts in their feces. Transmission modes include ingestion of sporulated oocysts – found in felid feces – or cysts found in muscular tissues of mammals or birds (intermediate hosts), and vertical transmission by tachyzoites that are passed to the fetus via the placenta [1, 4, 5].

*Toxoplasma* infection is prevalent in a large number of animal species, affecting even zoo and free-ranging animals [6–8]. In intermediate hosts, the prevalence may vary according to feeding habits, geographical region and species sharing the same ecosystem. *T. gondii* cysts in muscular tissues of wild animals are a potential source of infection for humans and other animals. Hunters and their families may also become infected during evisceration and game meat handling [9]. On the other hand, infected herbivorous indicates environmental contamination with oocysts, whereas infected omnivores indicate cumulative environmental contamination with oocysts and ingestion of infected muscular tissue of intermediate hosts [10]. Wild felids share some common areas in the environment with other wild animals, which allows the dissemination of the disease to the wild population.

In this study, a total of 26 run over or injured wild animals treated at the Dr. Halim Atique Veterinary Hospital of University Center of Rio Preto (UNiRP) – located in São José do Rio Preto, SP, Brazil (20°49′12″S, 49°22′44″W) – from June 2007 to August 2008 were investigated for *T. gondii* antibodies by the modified agglutination test (MAT), using a homemade formalin-fixed antigen and a cut-off titer of 10 [11–13]. The studied species were: *Alouatta caraya*, black howler monkey (n = 5); *Cebus apella*, tufted capuchin (n = 1); *Pseudalopex vetulus*, hoary fox (n = 6); *Cerdocyon thous*, crab-eating fox (n = 3); *Chrysocyon brachyurus*, maned wolf (n = 3); *Hydrochoerus hydrochaeris*, capybara (n = 4); *Puma concolor*, cougar (n = 2); *Leopardus pardalis*, ocelot (n = 1) and *Tapirus terrestris*, South American tapir (n = 1) (Table 1). The age of the animals was determined by examining their teeth.

**Table 1:** **Classification of wild animals investigated for** ***T. gondii*** **antibodies according to species, common name, gender, age, purpose of care and distribution per titer**

Scientific name	Common name	Sex (male/female)	Age (young/adult)	Purpose of care (run over/injured)	Titers (0/10/20/40)	Cities
*Alouatta caraya*	Black howler monkey	4/1	0/5	1/4	4/0/0/1	JB (3), SJRP (1), NH (1)
*Cebus apella*	Tufted capuchin	0/1	0/1	0/1	1/0/0/0	NH (1)
*Pseudalopex vetulus*	Hoary fox	3/3	2/4	6/0	5/1/0/0	JB (2), O (1), L (2), C (1)
*Cerdocyon thous*	Crab-eating fox	1/2	1/2	2/1	2/1/0/0	SJRP (1), N (1), P (1)
*Chrysocyon brachyurus*	Maned wolf	2/1	0/3	3/0	3/0/0/0	SJRP (1), J (2)
*Hydrochoerus hydrochaeris*	Capybara	2/2	0/4	2/2	3/1/0/0	SJRP (4)
*Puma concolor*	Cougar	2/0	0/2	2/0	1/0/0/1	N (2)
*Leopardus pardalis*	Ocelot	0/1	0/1	1/0	1/0/0/0	SJRP (1)
*Tapirus terrestris*	South American tapir	1/0	0/1	1/0	0/1/0/0	SJRP (1)

JB: José Bonifácio; SJRP: São José do Rio Preto; NH: Novo Horizonte; O: Olímpia; L: Lorena; C: Catanduva; N: Nhandeara; P: Promissão; J: Jales.

The hospital received wild animals found by the Federal Police on highways in São José do Rio Preto and other cities of São Paulo state including: José Bonifácio (21°03′10″S, 49°41′16″W), Novo Horizonte (21°28′04″S, 49°13′15″W), Olímpia (20°44′13″S, 48°54′54″W), Lorena (22°43′51″S, 45°07′30″W), Catanduva (21°08′16″S, 48°58′22″W), Nhandeara (20°41′38″S, 50°02′16″W), Promissão (21°32′13″S, 49°51′28″W) and Jales (20°16′08″S, 50°32′45″W).

The association between epidemiological data and serology results was analyzed using chi-square or Fisher’s exact tests, considering the significance level (α) of 5% [14]. All tests were processed by Epi Info™ v. 3.5.1. software [15].

*T. gondii* antibodies were detected in six of 26 (23.1%; CI95% 11.1-42.2%) wild animals, and titers were equal to 10 (4; 66.7%) and 40 (2; 33.3%). As to species, *Pseudalopex vetulus* (1), *Cerdocyon thous* (1), *Hydrochoerus hydrochaeris* (1) and *Tapirus terrestris* (1) had titters equal to 10, while *Alouatta caraya* (1) and *Puma concolor* (1) had titers equal to 40 (Table 1). Out of positive animals, three of six (50%) were found in São José do Rio Preto, while one specimen was found in Novo Horizonte, another one in Lorena and another one in Nhandeara. Among the three animals found in São José do Rio Preto, only one was carnivorous, *C. thous*, whereas the other two were herbivorous, *H. hydrochaeris* and *T. terrestris*. There was no significant difference as to gender, 5/15 (33.3%) were male and 1/11 (9.1%) female (*p* = 0.17). Similarly, considering age, there was no significant difference, 1/3 (33.3%) were young and 5/23 (21.7%) adult (*p* = 0.56) (Table 2). The purpose of care corresponded to 3/18 (16.7%) run over and 3/8 (30.5%) injured animals.

**Table 2:** **Association between epidemiological data and investigation for** ***T. gondii*** **antibodies in the studied animals**

Variable	N	MAT ^a^	Variable (%); CI95% ^b^	OR (CI95%) ^c^	***p*** ^d^
Gender					
Male	15	5	33.3; 15.2-58.7	5.0 (0.5-50.8)	0.17
Female	11	1	9.1; 2.1-38.5		
Age					
Young	3	1	33.3; 6.8-80.6	1.8 (0.1-24.2)	0.56
Adult	23	5	21.7; 9.8-42.2		
Purpose of care					
Run over	18	3	16.7; 6.1-39.6	3.0 (0.5-19.9)	0.25
Injured	8	3	30.5; 13.7-70.1		

^a^Titer ≥ 10.

^b^Frequency of positive animals by modified agglutination test (MAT) based on the studied variables (confidence interval 95%).

^c^OR: Odds ratio.

^d^ *p* value for significance level (α) of 5%.

The positive *C. thous* was a young male found injured on a highway in São José do Rio Preto (1/3, 33.3%). This prevalence of *T. gondii* antibodies was lower than that obtained by Gennari *et al*. [16] and Curi *et al*. [17], who found 60% (9/15) and 68% (13/19) positive results in free-range *C. thous* in São Paulo and Minas Gerais states, respectively. The crab-eating fox is considered an important sentinel for *T. gondii* infection in humans due to the high prevalence found in the aforementioned studies, mainly when environmental contamination by feces of felids was present. In the present study, another carnivore, *P. vetulus*, had one specimen positive (16.7%) that was run over in Lorena.

As to the data of the six studied non-human primates (five *Alouatta caraya* and one *Cebus apella*), only one *A. caraya* (1/6, 17%) was positive. It was a male adult that was injured in Novo Horizonte and had titer equal to 40. The same prevalence was obtained by Garcia *et al*. [18] in a study involving black howler monkeys *A. caraya* (3/17, 17.6%) and tufted capuchins *Cebus* spp. (13/43, 30.2%) in Paraná River Basin, Paraná state. The habitats of *Alouatta* and *Cebus* species are arboreal and terrestrial, respectively, and geophagy has been reported as their mineral source [19]. In this case, geophagy must be considered a relevant variable since these hosts are wild animals, which increases the possibility of infection, since they have more opportunity to be in contact with infective sources of *T. gondii* in natural environments where definitive hosts may live.

The only studied *Tapirus terretris*, Brazilian tapir, tested positive with titer equal to 10. This was a male adult animal, living in São José do Rio Preto and was taken to the university because it was run over. Infected tapirs are sources of infection for jaguars and cougars that prey on them [20].

The positive capybara (1/4, 25%), *Hydrochoerus hydrochaeris*, was a female adult that was injured in São José do Rio Preto. Similarly, Truppel *et al*. [21] observed 16/26 (61.5%) positive capybaras for *T. gondii* antibodies from Paraná state, and Yai *et al*. [22] found 49/64 (76.6%) positive animals in São Paulo state. The high percentage of this infection in Brazilian capybaras suggests a widespread environmental contamination with oocysts. These animals have great importance for toxoplasmosis epidemiology and have been considered sentinels for human infection since they are hunted for meat consumption. As capybara meat is consumed by human populations in different areas of South America, it may act as a source of infection by carrying *T. gondii* cysts. Hunters and their families may also become infected during evisceration and game meat handling [9].

Neotropical wild felids play an important role on the environmental maintenance of *T. gondii* oocysts and, if preyed, can be a source of infection for their predators. Although *Leopardus pardalis* was negative for *T. gondii* antibodies in the present study, Minervino *et al*. [20] reported 100% (3/3) positive animals in different places from Brazil. Thus, even with negative results, *L. pardalis* is suggested as having some importance in the transmission of toxoplasmosis in Brazil. A specimen of *Puma concolor* presented a high titer (40) whereas the other tested negative. The positive animal was a male adult that was run over and lived in Nhandeara. Kikuchi *et al.* [23] detected 98/438 (22.4%) positive free-range *P. concolor* throughout America and 21/59 (31.6%) only in South America.

Additionally, a large number of wild animals are considered sentinels for toxoplasmosis, including armadillos, coatis and marsupials [1]. The presence of Neotropical felids may facilitate the infection of these animals, since their feces comprise the main source of transmission to herbivores and omnivores. Further studies on the epidemiology of toxoplasmosis are required, mainly in wild animals, which confirms the importance of the present study.

## Conclusions

The present results demonstrate the importance of wild animals as sentinels of toxoplasmosis. In addition, this study reassures that the presence of wild felids comprises a risk for public health due to the maintenance of the environmental contamination.

### Ethics committee approval

The present study was approved by the Brazilian Institute of Environment and Renewable Natural Resources (IBAMA), license 218/2004-CGFAU/LIC, process 02027.002705/2000-05.

## References

[1] Acha PN, Szyfres B (2003). Zoonosis y enfermedades transmisibles communes al hombre y a los animals. Volume 3. 3ª edition.

[2] Weiss LM, Dubey JP (2009). Toxoplasmosis: A history of clinical observations. Int J Parasitol.

[3] Cenci-Goga BT, Rossitto PV, Sechi P, McCrindle CM, Cullor JS (2011). *Toxoplasma* in animals, food, and humans: an old parasite of new concern. Foodborne Pathog Dis.

[4] Marques JM, Da Silva DV, Correia NAB, Velásquez LG, Da Silva RC, Langoni H, Da Silva AV (2008). Prevalence and risk factors for human toxoplasmosis in a rural community. J Venom Anim Toxins incl Trop Dis.

[5] Langoni H, Modolo JR, Pezerico SB, Silva RC, Castro APB, Da Silva AV, Padovani CR (2006). Serological profile of anti-*Toxoplasma gondii* antibodies in apparently healthy dogs of the city of Botucatu, São Paulo State, Brazil. J Venom Anim Toxins Incl Trop Dis.

[6] Ullmann LS, Da Silva RC, De Moraes W, Cubas ZS, dos Santos LC, Hoffmann JL, Moreira N, Guimaraes AM, Montano P, Langoni H, Biondo AW (2010). Serological survey of *Toxoplasma gondii* in captive neotropical felids from Southern Brazil. Vet Parasitol.

[7] Zhang SY, Wei MX, Zhou ZY, Yu JY, Shi XQ (2000). Prevalence of antibodies to *Toxoplasma gondii* in the sera of rare wildlife in the Shanghai Zoological Garden. People’s Republic China Parasitol Int.

[8] Mucker EM, Dubey JP, Lovallo MJ, Humphreys JG (2006). Seroprevalence of antibodies to *Toxoplasma gondii* in the Pennsylvania bobcat (*Lynx rufus rufus*). J Wildl Dis.

[9] Dubey JP (1991). Toxoplasmosis–an overview. Southeast Asian J Trop Med Public Health.

[10] Dubey JP, Jones JL (2008). *Toxoplasma gondii* infection in humans and animals in the United States. Int J Parasitol.

[11] Desmonts G, Remington JS (1980). Direct agglutination test for diagnosis of *Toxoplasma* infection: method for increasing sensitivity and specificity. J Clin Microbiol.

[12] Dubey JP (2009). Toxoplasmosis of animals and humans.

[13] Smith DD, Frenkel JK (1995). Prevalence of antibodies to *Toxoplasma gondii* in wild mammals of Missouri and east central Kansas: biologic and ecologic considerations of transmission. J Wildl Dis.

[14] Triola MF (2005). Introdução à estatística.

[15] Center for Disease Control and Prevention (CDC) (2002). Epi Info [computer program]. Version 3.5.1.

[16] Gennari SM, Canon-Franco WA, Yai LE, De Souza SL, Santos LC, Farias NA, Ruas J, Rossi FW, Gomes AA (2004). Seroprevalence of *Toxoplasma gondii* antibodies from wild canids from Brazil. Vet Parasitol.

[17] Curi NA, Araújo A, Campos F, Lobato Z, Gennari S, Marvulo M, Silva J, Talamoni S (2010). Wild canids, domestic dogs and their pathogens in Southeast Brazil: disease threats for canid conservation. Biodivers Conserv.

[18] Garcia JL, Svoboda WK, Chryssafidis AL, de Souza ML, Shiozawa MM, de Moraes AL, Teixeira GM, Ludwig G, da Silva LR, Hilst C, Navarro IT (2005). Sero-epidemiological survey for toxoplasmosis in wild New World monkeys (*Cebus* spp.; *Alouatta caraya*) at the Parana river basin, Parana State, Brazil. Vet Parasitol.

[19] Auricchio P (1995). Primatas do Brasil.

[20] Minervino AH, Soares HS, Barreto-Junior RA, Neves KA, Pena HF, Ortolani EL, Dubey JP, Gennari SM (2010). Seroprevalence of *Toxoplasma gondii* antibodies in captive wild mammals and birds in Brazil. J Zoo Wildl Med.

[21] Truppel JH, Reifur L, Montiani-Ferreira F, Lange RR, de Castro Vilani RG, Gennari SM, Thomaz-Soccol V (2010). *Toxoplasma gondii* in capybara (*Hydrochaeris hydrochaeris*) antibodies and DNA detected by IFAT and PCR. Parasitol Res.

[22] Yai LE, Ragozo AM, Aguiar DM, Damaceno JT, Oliveira LN, Dubey JP, Gennari SM (2008). Isolation of *Toxoplasma gondii* from capybaras (*Hydrochaeris hydrochaeris*) from São Paulo State. Brazil J Parasitol.

[23] Kikuchi Y, Chomel BB, Kasten RW, Martenson JS, Swift PK, O’Brien SJ (2004). Seroprevalence of *Toxoplasma gondii* in American free-ranging or captive pumas (*Felis concolor*) and bobcats (*Lynx rufus*). Vet Parasitol.

